# Cholera Infantum

**Published:** 1880-11-20

**Authors:** Dudley M. Culver

**Affiliations:** Indiana


### "CHOLERA INFANTUM."

BY DUDLEY M. CULVER, M.D., INDIANA.

The season of the year is now at hand in which we are called upon to treat the little ones for bowel troubles, and upon this subject I wish to make some remarks. And, in writing on "cholera infantum," or any disease, I don't wish to be understood as assuming that I have a "cure-all." But my object is simply to give my best plan of treatment, and the reasons therefor founded upon notes of a series of cases.

Cholera infantum is so nearly allied to "cholera morbus" in adults, that I consider them as due to the same predisposing cause, but differing only in the exciting cause. Considering the extreme sensibility of the infant's nervous system and weakness of its apparatus, it is not surprising that such diseases should be prevalent, especially when we have the miasmatic influence to contend with. Among the exciting causes only two will be mentioned at this time, viz: dentition and indigestion. Dentition, if present, must, as a necessity, be relieved by scarification of the gums. Don't split the gums at a single stroke, as is practiced by unscrupulous men, for the incision will only heal at once, leaving a hard cicatrix, making the matter worse than if left alone. But simply scratch or scarify the gums sufficient to make them fester. Next turn your attention to the digestive organs; if indigestion is the trouble, diarrhœa is the consequence. Assimilation can't be performed in consequence of indigestion, and in turn digestion can't take place in consequence of non-assimilation. Now we find the little patient with fever, vomiting and diarrhœa. What must be done? The bowels are being irritated with stringy mucus and foul secretions. To check the bowels in this condition would only "add fire to a burn" by increasing the irritation and the fever. For this cause I always commence treatment with a full dose of castor oil. This clears the whole alimentary canal of the irritating ingesta, and places them in a condition that the diarrhœa may be checked with perfect impunity.

To check the diarrhœa after the operation of the oil, nothing answers my purpose so well as tannic acid in two-grain doses in solution, (after each operation on the bowels); it makes a perfect solution in water. For the vomiting, an admirable treatment is strong coffee without milk or sugar in teaspoonful doses, repeated when necessary, with a poultice of peppermint (bruised peppermint leaves) laid on the stomach. In addition to the above, I invariably give small doses of quinine in solution in nitrous ether. It answers the purpose of being a good febrifuge and an anti-miasmatic, both of which are an absolute necessity with an eye to success. It makes me shudder when I think of the laudanum internally and the large fly-blisters externally that have been applied to the treatment of this malady. We should be humane enough to remember that we are treating a child, and the milder our treatment the better.—*Obstetric Gazette.*

